# Harnessing Macrophages for Controlled-Release Drug Delivery: Lessons From Microbes

**DOI:** 10.3389/fphar.2019.00022

**Published:** 2019-01-25

**Authors:** Johan Georg Visser, Anton Du Preez Van Staden, Carine Smith

**Affiliations:** Department of Physiological Sciences, Stellenbosch University, Matieland, South Africa

**Keywords:** synthetic microbe, expulsion, cell-mediated delivery, biopharmaceutical, phagocytosis, intracellular pathogen, polymer, nano-medicine

## Abstract

With the effectiveness of therapeutic agents ever decreasing and the increased incidence of multi-drug resistant pathogens, there is a clear need for administration of more potent, potentially more toxic, drugs. Alternatively, biopharmaceuticals may hold potential but require specialized protection from premature *in vivo* degradation. Thus, a paralleled need for specialized drug delivery systems has arisen. Although cell-mediated drug delivery is not a completely novel concept, the few applications described to date are not yet ready for *in vivo* application, for various reasons such as drug-induced carrier cell death, limited control over the site and timing of drug release and/or drug degradation by the host immune system. Here, we present our hypothesis for a new drug delivery system, which aims to negate these limitations. We propose transport of nanoparticle-encapsulated drugs *inside* autologous macrophages polarized to M1 phenotype for high mobility and treated to induce transient phagosome maturation arrest. In addition, we propose a significant shift of existing paradigms in the study of host-microbe interactions, in order to study microbial host immune evasion and dissemination patterns for their therapeutic utilization in the context of drug delivery. We describe a system in which microbial strategies may be adopted to facilitate absolute control over drug delivery, and without sacrificing the host carrier cells. We provide a comprehensive summary of the lessons we can learn from microbes in the context of drug delivery and discuss their feasibility for *in vivo* therapeutic application. We then describe our proposed “synthetic microbe drug delivery system” in detail. In our opinion, this multidisciplinary approach may hold the solution to effective, controlled drug delivery.

## Introduction

In recent years, drug delivery has become a well-documented research niche across various disciplines in science. Approaches of drug delivery into pathogenically damaged areas or poorly vascularised cancer tissues has been largely focused on treatments incorporating nanoparticles (Zhao et al., 2011; Dreaden et al., 2012; Feng et al., 2014; Huang et al., 2015; Lv et al., 2016; Tanei et al., 2016). These nanoparticles generally serve to shield harsh/labile drugs from the host and subsequently activate or release it after reaching target tissues. With the potential exception of nanoparticle uptake into target cells via complementary receptor ligands, this approach is, however, still more comparable to drug saturation than with specialized drug delivery *per se*.

In this review we propose an alternative to the strategies/approaches used until now: a novel macrophage-mediated drug delivery method that more accurately fits the term “drug delivery,” via incorporation of both nanomedicine and cellular manipulation. Macrophages are highly mobile cells. By loading host macrophages with appropriate “cargo” (e.g., chemotherapeutic agents such as doxorubicin or high-potency antimicrobials), one can thus theoretically use the inherent homing capabilities of these immune cells to reach target damaged, infected or malignant tissue, in order to treat the affected cellular areas only. Such an approach would reduce the total concentration of drug required (when compared to systemic administration) and significantly reduce or even eradicate the risk of drug-associated adverse effects. Achieving this goal would indeed require substantial research into phagocytosis, macrophage chemotaxis, pathogenic immune evasion and controlled release of therapeutics. Here, we propose such a system where “cargo” is introduced into the macrophage, maintained within “inactivated” phagosomes and released in a controlled manner at the appropriate time and *in vivo* location.

The system as proposed in its entirety here, is novel. However, some aspects of this system have been investigated individually before (discussed in detail in Section “The Impossible Made Possible?”) and testifies to the feasibility of the approach we suggest. In order to fully understand cellular role players, a multidisciplinary approach is clearly required. We propose that the literature on host–microbe interactions may provide the insight required. While research has described the ability of microbes to evade the immune system by hiding (and proliferating) inside immune cells before orchestrating their own expulsion or transfer directly into new host cells, the mechanisms by which they achieve this have received very little attention by non-microbiologists. In our opinion, harnessing these microbial strategies could prove useful in the drug delivery niche. Thus, if a paradigm shift can be made to embrace the fact that host-affecting microbial mechanisms may potentially have therapeutic application, we believe that biologists could learn valuable lessons from microbes, to the benefit of technological advancement in medicine.

The aim of this paper is therefore to present a summary of pertinent literature on microbial mechanisms known to modulate the course of endocytic processes and to evaluate their feasibility in the context of therapeutic drug delivery. A specific novel focus will be on potential mechanisms through which to achieve controlled expulsion. We believe that this paper elucidates an exciting new avenue for research in the context of drug delivery.

In order to facilitate clarity of our argument, we first provide a brief overview of the most pertinent literature describing the mechanisms that would come into play in a complete cell-based delivery system. Considering the complexity of these processes, one can appreciate the enormity of the task to elucidate which perturbations in this process may be used for application to our proposed drug delivery system. Thus, we will describe the different phases – namely cargo loading, maintenance of cargo integrity, *in vivo* motility of the carrier cell toward delivery sites and cargo expulsion – individually below, before discussing in more detail, the lessons to be learnt from microbes.

## Components of a Cell-Based Delivery System

### Cargo Loading Into Macrophages

**FIGURE 1:**
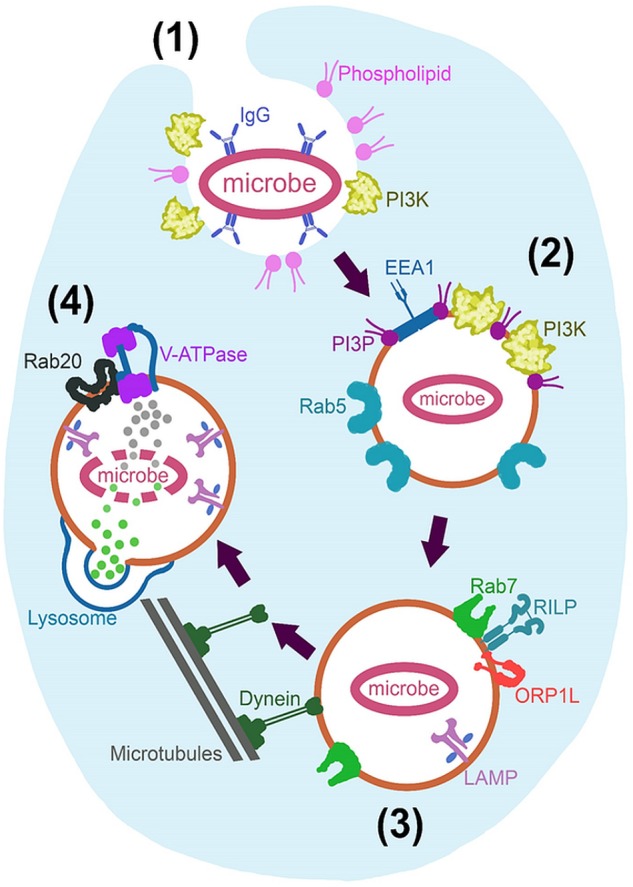
Fundamental mechanisms of phagosome maturation. Initiated through (1) Recognition and engulfment of opsonised microbe and expression of phospholipids and phosphoinositide 3-kinase (PI3k), at the extending pseudopodia. (2) Nascent phagosome is formed after actin polymerization facilitates pseudopod closure behind the microbe. This phagosome is characterized by Rab5, phosphatidylinositol 3-phosphate (PI3P) and endosomal early antigen 1 (EEA1) expression. (3) The late phagosome is characterized by Rab7 recruitment; resulting in Rab5 inactivation and PI3P degradation as well as recruitment of lysosome-associated membrane proteins (LAMP) while achieving dynein linkage and centripetal movement for later lysosomal fusion. Rab7 achieves these processes via Rab7-interacting-lysosomal-protein (RILP) and oxysterol-binding protein related-protein 1 (ORP1L). Lysosome fusion initiates the last stage in maturation; (4) Phagolysosome biogenesis, where LAMP expression is increased and lysosomal content is dumped into the phagosome. Rab20 also allows an acidic environment through the action of vacuolar-type H^+^-ATPase (V-ATPase).

Circulating monocytes form part of the innate immune system and are largely responsible for the initial recognition of foreign material or microbes (Abbas et al., 2014). Recognition and internalization, for the purpose of neutralization, are generally very effective. This is evidenced by the absence of adaptive B and T cell responses in almost 95% of *Animalia* (Mills et al., 2015). However, many microbes have been able to survive within macrophages by manipulating phagocytic processes (discussed later in Section “What Can We Learn From Microbes?”). A summary of the most relevant normal human phagocytic processes is presented visually in Figure 1.

The most important aspect of our topic, is that of immune recognition and uptake into the macrophage. It is commonly known that pattern recognition receptors (PRRs) on phagocytes recognize several different molecular patterns – such as damage-associated molecular patterns (DAMPs) or pathogen-associated molecular patterns (PAMPs) – on the target for potential phagocytosis (Abbas et al., 2014). Toll-like receptors (TLRs) present on phagocytes also indirectly regulate phagocytosis through Myeloid differentiation primary response 88 (Myd88) signaling and activation of the p38 residue (Shi et al., 2016). Several other minor role players in pathogen recognition, such as receptors for lectin, mannose, complement and Retinoic acid-inducible gene-I-like (RIG-like) receptors, have been identified, but the immunoglobulin G (IgG) receptors are most directly associated with phagocytosis of material. In fact, antibody-opsonised material binds and activates IgG receptors to induce engulfment independently of co-stimulation by T cells or NK (natural killer) cells (Liu et al., 2013), making this mechanism an obvious choice for *ex vivo* cargo loading into macrophages. Engulfment is reliant on phosphoinositide 3-kinase (PI3k) recruitment and its production of various phosphatidylinositides that, together with actin polymerization, result in pseudopod formation around the material and subsequent internalization.

Once material has been engulfed, it is enveloped inside a double-membraned (nascent) phagosome, which is innocuous and undergoes various maturation phases, that culminates in fusion with lysosomes, which enables it to acidify and break down its contents. Characterization of the engulfment and early maturation processes is well-established (Patki et al., 1998; Fratti et al., 2001; Vieira et al., 2002, 2003; Kinchen et al., 2008; Fairn and Grinstein, 2012) and are not discussed in more detail here, as we do not envisage a requirement for huge manipulation of this phase. Indeed, previous research by our group and others have demonstrated that macrophages readily take in a variety of purpose-designed materials and particles of varying sizes via endocytic pathways (Muthana et al., 2011; Zhao et al., 2011; Dreaden et al., 2012; Feng et al., 2014; Oh and Park, 2014; Huang et al., 2015; Miller et al., 2015; Tanei et al., 2016; Fan et al., 2018; Visser and Smith, 2018).

### Cargo Maintenance

Of more direct relevance, lysosomal fusion marks the start of the last stage in maturation, that of phagolysosome biogenesis (Seto et al., 2011), which is an obvious threat to cargo maintenance. Normally, this lysosome fusion is mediated by endoplasmic reticulum (ER) soluble *N*-ethylmaleimide-sensitive factor-attachment proteins (SNARE) such as syntaxin 7, syntaxin 8 and vesicle associated membrane protein (VAMP) -7 and -8 (Becken et al., 2010). Lysosome-associated membrane protein (LAMP) concentration is increased after fusion (Jahraus et al., 1994) and cathepsin D proteases are recruited from the Golgi via Rab-22b, -32, -34, -38, and -43 (Ng et al., 2007). The vacuolar-type H^+^-ATPase (V-ATPase) is also incorporated via Rab20 co-localization at this time (Curtis and Gluck, 2005). In this way, fusion ultimately effectuate an acidic environment within the macrophage phagosome, as well as supplying it with proteases, reactive oxygen species (ROS) and reactive nitrogen species (RNS) to facilitate decomposition of phagosomal content.

To date, the majority of literature employing macrophages as delivery shuttles, have used either nanoparticle-encapsulated drugs traveling inside the cell, or drugs “backpacked” on the outside of the cell. The most popular protocol used is loading cargo into macrophages to create a “Trojan horse.” However, this approach has some limitations: firstly, there is a significant risk of drug-associated cytotoxicity, secondly, drugs are released at a relatively slow rate and thirdly, they are vulnerable to lysosomal degradation inside the macrophage (Yousefpour and Chilkoti, 2014). In an attempt to address these limitations, transport of drugs on the outer surface of macrophages were attempted. However, prevention of internalization of the backpacked cargo into carrier macrophages was a major obstacle (Holden et al., 2010; Doshi et al., 2011).

In our opinion, perhaps the most feasible option to ensure integrity of cargo that are either labile or highly toxic – so that premature delivery should not be risked – would be their maintenance intracellularly by modification of normal phagocyte function. It is here where we could substantially learn from microbial strategies (refer to Section “What Can We Learn From Microbes?”). Indeed, we have previously demonstrated maintenance of cargo inside primary human M1 macrophages chemically treated to transiently inhibit phagosomal cargo destruction (Visser and Smith, 2018). Briefly, protein-coated polystyrene beads, used as simulative cargo, were maintained intact (i.e., with no digestion of the protein coating) inside macrophages after *in vitro* treatment with a phagosome maturation inhibiting cocktail, consisting of Wortmannin, Concanamycin A and Chloroquine. This inhibition cocktail was only administered *in vitro*, and treated cells were washed prior to use, thus lowering risk to patient in the context of *in vivo* application. Furthermore, this intervention did not affect chemotactic or migratory capacity – macrophages were able to transverse an *in vitro* Human Umbilical Vein Endothelial Cell (HUVEC) membrane while carrying the bead cargo.

Another modern technique relevant here, is the use of nano- or microparticle encapsulation of drugs prior to loading into carrier cells (Dou et al., 2006; Zhao et al., 2011; Chang et al., 2013; Blaudszun et al., 2014; Feng et al., 2014; Gnanadhas et al., 2017; Klyachko et al., 2014; Pang et al., 2016; Tanei et al., 2016; Evans et al., 2018; Fan et al., 2018). In addition to host protection, polymeric particles may also be used for maintenance of drug integrity itself. Emerging evidence indeed indicates a role for polymeric particles as protective modality for both host and drug cargo. Cargo can be rendered innocuous via, for example, poly-(NIPA-co-AAm) (PNIPAAm) micelle or microbubble encapsulation. PNIPAAm micelles are reported to degrade in response to an increase in temperature above the lower critical solution temperature (Feng et al., 2014), enabling control over bio-activation of encapsulated drugs. As an example, these micelles could be incorporated during treatment of peripheral diseases, such as melanoma or myopathy, where an external stimulus can be administered to increase the local temperature and release drug cargo from PNIPAAm micelles. Incorporation of microbubbles together with nanoparticles has also shown some promise during *in vivo* delivery of resveratrol for treatment of cancer (Lv et al., 2016). In this study, resveratrol was loaded into acetylated β-cyclodextrin nanoparticles (PNP), which were then loaded into microbubbles. The outer microbubble coating served to protect the pH sensitive PNP while in circulation, whereas PNP in turn released resveratrol upon exposure to the low pH tumor niche. These studies indicate that polymeric particles may be powerful tools to incorporate into delivery systems to address current limitations.

### *In vivo* Macrophage Migration for Cargo Delivery

Literature focusing on macrophage (or other phagocyte) migration are normally aimed at the prevention of this migration, e.g., in the context of cancer metastasis or inflammation. Despite the different focus to ours, these studies have elucidated the process of migration in detail.

For example, in the context of muscle inflammation, M1 macrophages have been illustrated to be the most motile, pro-inflammatory phenotype, while M2 macrophages are less likely to infiltrate tissues and associated with a relatively anti-inflammatory outcome (Arnold et al., 2007; Smith et al., 2008; Kruger et al., 2014). Our previous work on M1 macrophages (Visser and Smith, 2018) confirms the choice of this phenotype as superior for drug delivery. However, it should be noted that macrophage phenotype has a large degree of plasticity, which will have to be taken into account when designing drug delivery protocols for application in particular disease states. To this end, a recent review (Ruytinx et al., 2018) comprehensively provide information on macrophage polarization in the context of inflammatory diseases such as neurological disease, cancer, metabolic and cardiovascular disorders.

Similarly, in terms of chemotactic signals for macrophage migration, chemotaxins generally expressed on tissue cells in many different disease conditions have been identified. Most notably, inflammation – which would be present in any disease state with a requirement for drug delivery – is known to result in increased levels of the chemokines macrophage migration inhibitory factor (MIF) and/or macrophage chemotactic protein (MCP-1 or CCL2), which are strong signals for macrophage recruitment into the tissue (Lee et al., 2010; Baeck et al., 2012). Additionally, oxidative stress – which is a known complication of both chronic disease and infection (Nimse and Pal, 2015; Petersen and Smith, 2016) – have been shown to initiate macrophage migration *in vivo* (Wang et al., 2016). In terms of systemic migration toward chemotactic signals originating from hypoxic tissue, such as in cancer, evidence also exist to confirm the inherent capacity of macrophages to migrate toward tumors (Owen et al., 2004; Batrakova et al., 2011). Although finer detail on the regulation of macrophage migration has been reported, such as its dependence on integrin β1 expression and recycling (Lee et al., 2017) and numerous proteases (Van Goethem et al., 2018), these details are likely of academic value only, at least for the context under discussion. In our opinion, these factors are unlikely to be a limiting factor, since integrin β1 is expressed by almost all cell types and some degree of redundancy is in place. For example, in contrast to the fairly uniform amoeboid movement of neutrophils, macrophages were reported to exhibit multiple different migration mechanisms that are more mesenchymal in nature (Barros-Becker et al., 2017; Van Goethem et al., 2018), which could confer greater resilience to macrophages in terms of mobility under adverse conditions. Interestingly, the latter study also demonstrated a larger degree of directionality of macrophages *vs.* neutrophils in a zebrafish tail transection model of leukocyte migration. Another ability of macrophages pertinent to the current topic, is their ability to maintain their mobility after ingestion of cargo, even when the cargo is much larger than anticipated to be required for the purpose of drug delivery (Chang et al., 2013; Evans et al., 2018; Visser and Smith, 2018). These reports again confirm that this phase of the system is unlikely to require major intervention, as it seems to already have been fine-tuned by evolution.

The only potential limitation we foresee is interference with chemotactic signal originating from the intended site for drug delivery, by e.g., an acute, severe infection/damage in another organ, which may have chemotactic priority above that of the signal originating from the intended delivery site. However, the practice of isolating patients for a period prior to a medical procedure is not uncommon and could avoid this complication. Furthermore, pathogen-associated infection has been shown to take priority above other, relatively less life-threatening, situations, which would in fact favor directional macrophage migration, rather than limit it.

### Cargo Expulsion

The final step to complete such a delivery system, would be a mechanism by which the cargo can be released or expelled at the appropriate time and location *in vivo*. Normally, following phagolysosomal destruction of ingested material, digested material is either recycled by the phagocytic cell or expelled into the extracellular matrix. Recycling of re-usable “waste” such as amino acids, glucose and phosphates occur via diffusion through the phagolysosome membrane into the cytosol (Guyton and Hall, 2011). Of particular interest here, the insoluble components are expelled from the macrophage either via the ER-Golgi secretory pathway or utilized for antigen presentation through Ca^2+^- and vesicle-associated membrane protein 7 (VAMP7)-dependant lysosome exocytosis (Samie and Xu, 2014). We believe that the manipulation of these expulsion mechanisms could facilitate controlled drug delivery.

In terms of published studies on drug delivery systems, most systems either rely on non-specific release of nanoparticles containing drugs (Miller et al., 2015), or employ release of drugs inside the carrier cell. For example, rupture of doxorubicin-containing microbubbles inside macrophages was achieved by high intensity focused ultrasound techniques (Fan et al., 2018). However, this strategy for drug release resulted in significant carrier cell death. We believe this is an undesirable mechanism, as this would likely contribute to inflammation and thus delayed recovery. Therefore, to date, a controlled cell-based drug release system does not seem to exist.

In the next section, we evaluate microbially employed strategies, in terms of their feasibility for adaptation into therapeutic contexts. We will focus on the two phases of this system which seems to be most commonly and effectively manipulated by microbes, namely immune evasion by intracellular survival and programmed expulsion from host cells.

## What Can We Learn From Microbes?

Pathogenic phagosome maturation arrest or modulation thereof, and subsequent excape from the host cell are hallmarks of bacterial host immune evasion and dissemination. Well characterized mechanisms include interfering with PI3k and PI3P biogenesis (*Mycobacterium tuberculosis* and *Candida glabrata*) (Vergne et al., 2003; Rai et al., 2015), establishing microbe-containing vacuoles (*Legionella pneumophila* and *Brucella*) (Celli, 2015; Bärlocher et al., 2017), blocking of fission and fusion with lysosomes and endosomes (*Mycobacterium tuberculosis* and *Legionella*) (Vergne et al., 2003, 2005; Bärlocher et al., 2017), raising pH levels via induction of phagosomal acid leakage (*Cryptococcus neoformans*) (Tucker and Casadevall, 2002), lysis of the phagosomal membrane (*Listeria monocytogenes*) (Alberti-Segui et al., 2007), hijacking of the endocytic recycling pathway (*Legionella pneumophila*) (Xu and Luo, 2013) and even active macrophage killing (filamentous *Candida albicans*) (Gaur et al., 2013). Manipulation of the endocytic pathways by microbes is achieved via highly diverse and complex mechanisms. Intracellular microbes secrete hundreds of proteins, known as effectors, capable of modulating these pathways (Santos et al., 2015; Schroeder, 2018). These effectors have diverse functions and microbes employ multiple layers of redundancy to ensure their survival (Ghosh and O’Connor, 2017; Schroeder, 2018). The abundance and variety of these effectors provide an ideal bioprospecting opportunity to identify effectors that can be utilized to modulate the endocytic pathways as needed. Keeping in mind that not all microorganisms have effectors capable of manipulating the endocytic pathway for both maintenance and expulsion. Identified effectors can then be further investigated to optimize the cocktail of effectors (possibly from different organisms) best suited for application in a macrophage-based delivery system.

Examples of intracellular microbes and their mechanisms for modulation of the endocytic pathway are summarized in Table 1. In order to provide more detail on the variety and complexity of methods used, modulatory mechanisms of different microbes in the context of both phagosome maturation and expulsion are presented in the next sections.

**Table 1:** Examples of intracellular microbes and main outcomes of endocytic pathway modulation.

Organism	Disease state	Type of endocytic modulation	Outcome of endocytic modulation	Egress and dissemination
*Brucella* (G-) ^∗^ 1	Brucellosis/Malta Fever	Modulation of phagosome maturation	VirB (T4SS) dependent modulation of phagosome, Suppression of macrophage polarization	Cell necrosis/apoptosis (VirB and bacterial dissociation dependent) followed by extracellular dissemination
*Legionella pneumophila* (G-) ^∗^ 2	Legionnaires disease	Prevention of phagosome maturation	Dot/Icm (T4BSS) dependent prevention of phagosome maturation	Pyroptosis, Apoptosis, Cell lysis
*Listeria monocytogenes* (G+) ^∗^ 3	Listeriosis	Prevention of phagosome maturation, phagosome rupture	Listeriolysin-O dependent lysis of phagosome or induction of autophagy for replication	Cell-to-cell spread (ActA/LLO-dependent)
*Chlamydia* (G-) ^∗^ 4	Genital/respiratory infections	Subversion of endocytic pathway via inclusion formation	Inc/CT229 dependent inclusion development	Cell lysis, Inclusion extrusion
*Mycobacterium tuberculosis* (acid fast) ^∗^ 5	Tuberculosis	Prevention of phagosome maturation, phagosome rupture	LAM/SapM dependent interference of PI3k and PI3P biogenesis. Inhibition of H+ V-ATPase assembly. ESX-1/EsxA and PDIM dependent phagosome rupture	Cell necrosis/apoptosis
*Salmonella enterica* (G-) ^∗^ 6	Salmonellosis	Modulation of phagosome maturation	Modulation of phagosome maturation via T3SS effector (SifA, SopB) dependent development of SCV	Pyroptosis
*Cryptococcus neoformans* (Yeast) ^∗^ 7	Cryptococcal meningitis	Unknown effectors. Possible pH dependent phagosome damage. Capsular protection.	Unknown effectors. Possible pH modulation through phagosome damage.	Non-lytic expulsion, cell lysis, cell-to-cell spread

*Gram negative (G-), Gram positive (G+). ^∗^1: Pizarro-Cerdá et al., 1998; Hong et al., 2000; Comerci et al., 2001; Boschiroli et al., 2002; Celli et al., 2003, Celli, 2006, 2015; Arellano-Reynoso et al., 2005; Pei et al., 2008, 2006, 2014; Starr et al., 2008, 2012; Chen and He, 2009; Chen et al., 2011; Von Bargen et al., 2012; Smith et al., 2016. 2: Kirby et al., 1998; Roy et al., 1998; Gao and Abu Kwaik, 1999; Alli et al., 2000; Gerhardt et al., 2000; Bachman and Swanson, 2001; Tilney et al., 2001; Molmeret et al., 2002, 2004; Chen et al., 2004, 2007; Xu and Luo, 2013; Schroeder, 2018. 3: Smith et al., 1995; Veiga and Cossart, 2005; Henry et al., 2006; Shaughnessy et al., 2006; Alberti-Segui et al., 2007; Birmingham et al., 2008; Czuczman et al., 2014; Mitchell et al., 2015; Skoble et al., 2017. 4: Perfettini et al., 2003; Scidmore et al., 2003; Rzomp et al., 2003; Hybiske and Stephens, 2007; Betts-Hampikian and Fields, 2010; Capmany and Damiani, 2010; Chin et al., 2012; Volceanov et al., 2014. 5: Sturgill-Koszycki et al., 1994; Ferrari et al., 1999; Renshaw et al., 2002; Walburger et al., 2004; Vergne et al., 2005; de Jonge et al., 2007; Seto et al., 2010, 2011; Wong et al., 2011; Simeone et al., 2015; Zhang et al., 2016; Augenstreich et al., 2017; Queval et al., 2017; Quigley et al., 2017. 6: Hersh et al., 1999; Steele-Mortimer et al., 1999; Jesenberger et al., 2000; Sano et al., 2007; Bujny et al., 2008; Mallo et al., 2008; Bakowski et al., 2010; Braun et al., 2010; McGourty et al., 2012; Chakraborty et al., 2015; D’Costa et al., 2015; LaRock et al., 2015; Li et al., 2016; Knuff and Finlay, 2017. 7: Wozniak and Levitz, 2008; Johnston and May, 2010; Nicola et al., 2011, 2012; Qin et al., 2011; Chen et al., 2015; Davis et al., 2015; Smith et al., 2015; Bojarczuk et al., 2016; Gilbert et al., 2017.*

### Intracellular Survival Mechanisms

Due to the high incidence of tuberculosis in especially developing countries, much research has been focused on the causative agents of this illness. As a result, relatively detailed knowledge is available on the route of immune evasion by this pathogen in particular, as well as on how bacterially secreted effectors and cell wall components modulate phagosome maturation. The primary route of *Mycobacterium tuberculosis* (Mtb) into the body is through inhalation, where it reaches the lungs’ alveolar space and is preferentially taken up by alveolar macrophages. Mtb survive intracellularly by working against PI3ks to prevent EEA1 docking. This is achieved in two ways: (1) Mtb secretes a PI 3′-phosphatase (SapM) that dephosphorylates PI3P and (2) a component in the microbial cell wall, lipoarabinomannan (LAM), interferes with recruitment/activation of the human PI3k (hVPS34) (Vergne et al., 2005). Mycobacterium-containing phagosomes also retain the tryptophan-aspartate containing coat (TACO) protein (normally expressed on the cytosolic leaflet of the plasma membrane and involved in intracellular membrane trafficking, cytokinesis and cytoskeletal remodeling) (Ferrari et al., 1999). TACO retention causes prolonged Rab5 expression – although some maturation effectors can still bind the phagosome, this effectuates a relative absence of PI3P, so that the FYVE domain-mediated binding of EEA1 is greatly perturbed (Simonsen et al., 1998) and lysosome fusion inhibited (Ferrari et al., 1999). Additionally, secretion of the soluble serine/threonine kinase Protein kinase G (PKG) by Mtb into the host cytosol is essential for prevention of phagosome-lysosome fusion (Walburger et al., 2004). Furthermore, a more alkaline and hydrolase deficient phagosome is also brought about in two ways. Firstly, hydrolysis is weakened by limited expression of Rab7. This GTPase has been shown to only transiently localize to mycobacterial phagosomes, preventing sufficient Rab7-interacting lysosomal protein (RILP) recruitment, but also limiting cathepsin D protease delivery (Seto et al., 2010, 2011). Secondly, acidification is regulated by Mtb by interfering with V-ATPase complex assembly and retention, thereby maintaining a stable, slightly alkaline pH (6.2–6.5) (Sturgill-Koszycki et al., 1994; Seto et al., 2011; Queval et al., 2017). The Mtb phosphatase, PtpA, is involved in inhibition of complex assembly by binding to the subunit H of the V-ATPase where it then dephosphorylates and inactivates hVPS33B, effectively inhibiting the membrane fusion machinery (Wong et al., 2011).

In contrast to Mtb, the survival mechanisms of *C. glabrata* is largely dependent on active PI3ks. *C. glabrata* encodes the enzyme PI3k and produces fungal PI3P (Strahl and Thorner, 2007; Rai et al., 2015). In this manner, the PI3P content of phagosomes increase prematurely during the early stages of maturation, where PI3P has not yet come into play. This could lead to a PI3P rich phagosome being identified as already partly matured, thus halting further maturation. Additionally, increased PI3P content could overburden PI3P degradation capacity of the phagosomal lumen. Deletion of the functional subunits of fungal PI3k led to ameliorated phagosome maturation and significantly reduced fungal survival and virulence (Rai et al., 2015). The differences between the strategy of *C. glabrata* vs. Mtb illustrates how the same cellular role players may be modulated in different ways for different outcomes, depending on the intended requirement of the modulating microbe, and in our opinion also demonstrates the susceptibility of this system to exogenous modulation or control.

Reminiscent of Mtb, *Leishmania* (the causative agent of Leishmaniasis) promastigotes are also harbored in phagosomes that retain TACO on their membranes, blocking lysosome fusion and ensuring a neutral pH in which this parasite can differentiate into the amastigote stage (Ferrari et al., 1999; Gogulamudi et al., 2015). However, after differentiation, the parasite allows phagosome fusion with lysosomes to achieve an acidic environment in which the amastigotes thrive. Interestingly, these phagosomes still exhibit low expression of late phagosomal markers (LAMP, V-ATPase and Rab7), after lysosome fusion (Vinet et al., 2009). In addition, *Leishmania* protects itself by inhibiting recruitment of NADPH oxidase to the phagosome, perturbing ROS production (Moradin and Descoteaux, 2012). Similarly, *M. tuberculosis* was reported to stimulate release of TNF-α and IL-10 from infected macrophages (Sendide et al., 2005), resulting in a deactivation of ROS and RNS release (Redpath et al., 2001). IL-10 specifically down-regulates secretion of pro-inflammatory cytokines (Redpath et al., 2001) like INF-γ and TNF-α and results in a shift toward a Th2-type cell expansion in the alveoli (de Almeida et al., 2012), bringing about a shift toward an alternatively activated, anti-inflammatory, M2 macrophage phenotype (Smith et al., 2008), which itself produces more IL-10, sustaining this phenotype and a relatively more anti-inflammatory environment. This implies that these microbes not only alter the response of the host cell to the ingested microbe itself, but that it may also affect systemic signaling by the host cell, which may affect the rate at which these bacteria are able to spread.

*Brucella* and *Legionella* are examples of intracellular pathogens that manipulate the endocytic pathway to create a niche in which they can replicate and thrive. They accomplish this by hijacking host proteins and membrane organelles to establish a bacterium-containing vacuole with morphological features reminiscent to that of host membrane compartments (Xu and Luo, 2013; Celli, 2015). Effectors secreted by *Brucella* within the *Brucella-*containing vacuole (BCV) manipulates maturation by altering interactions with late endosomes and lysosomes (Celli, 2015). During initial phagocytosis a large portion of *Brucella* cells are rapidly degraded (∼90%), however, surviving cells are capable of prolonged intracellular proliferation (Von Bargen et al., 2012). Previously it was thought that *Brucella* evade fusion of BCVs with lysosomes by secretion of effectors via a functional VirB type IV secretion system (T4SS) and cyclic β-1-2-glucan (Pizarro-Cerdá et al., 1998; Celli et al., 2003; Arellano-Reynoso et al., 2005). Cyclic β-1-2-glucan was thought to prevent fusion of the BCV with lysosomes by modulating lipid raft organization on phagosome membranes, but is not a requirement for subsequent BCV maturation (Arellano-Reynoso et al., 2005). Rather, live cell imaging has shown that the BCV interacts with lysosomes, thus fusion is not completely prevented (Starr et al., 2008). Early stages of BCV maturation involve the recruitment of late endosome markers, LAMP-1 and Rab7 to the BCV membrane, with acidification of the BCV being crucial for VirB expression (Boschiroli et al., 2002; Starr et al., 2008). This early BCV is also known as the endosomal BCV (eBCV) due to its interaction with the endocytic pathway. These findings highlight the importance of the initial interactions with the endocytic pathway in determining outcome. Unlike *Brucella, Legionella* diverts from the canonical endocytic pathway soon after being phagocytosed (Tilney et al., 2001). Departure from the canonical endocytic pathway starts minutes after being phagocytosed – the *Legionella-*containing vacuole (LCV) is covered with smooth vesicles, ER in origin, and mitochondria is recruited to the LCV (Tilney et al., 2001). The LCV is also devoid of early endosome markers such as Rab5 and LAMP-1, with the exception of Rab7 (Roy et al., 1998). *L. pneumophila* utilizes early mild caspase-3 activation to prevent lysosome fusion by cleavage of rabaptin5 (effector of Rab5) (Gao and Abu Kwaik, 1999; Molmeret et al., 2004). The eBCV and LCV both eventually interact with components of the ER. In the case of the eBCV, LAMP-1 is progressively lost as the eBCV interacts with the ER and maturation proceeds to the formation of a replication-permissive BCV (rBCV) (Celli et al., 2003; Celli, 2006; Starr et al., 2008), with the rBCV subsequently converted from an intermediate vacuole into an ER-derived organelle which is ideal for bacterial proliferation (Celli et al., 2003). The smooth vesicles recruited to the LCV early on, eventually come to resemble rough ER and become studded with ribosomes (Gerhardt et al., 2000; Tilney et al., 2001). The specific recruitment of GTPases usually required for fusion of ER-derived vesicles with the Golgi apparatus aids in this process. Similar to *Brucella*, the hijacking of the host’s secretory trafficking pathway results in a replication-permissive LCV. The rapid formation of an ER-like LCV and subversion of the endocytic pathway is dependent on the Dot/Icm T4BSS (Defective in Organelle Trafficking/Intra-Cellular Multiplication Type 4B Secretory System) of *Legionella* (Roy et al., 1998). Indeed, mutants deficient in the T4BSS ultimately fuse with the lysosome, indicating that effectors secreted by the T4BSS directly influence the endocytic pathway (Roy et al., 1998; Molmeret et al., 2004; Schroeder, 2018). The Dot/Icm T4BSS secretes hundreds of potential virulence effector molecules that aid in the formation of the LCV. However, no one effector has been shown to be crucial, again indicative of multiple layers of redundancy (Schroeder, 2018). Similarly, the VirB T4SS is essential for *Brucella* survival, as illustrated in *virB*–mutants (Hong et al., 2000; Comerci et al., 2001; Celli et al., 2003; Pei et al., 2008). Several other pathogens utilize T4SS and other secretion systems to release effector molecules that are capable of manipulating host function. The VirB and Dot/Icm systems are certainly also capable of releasing effector molecules that, in the case of *Brucella* and *Legionella*, are used to manipulate ER membrane dynamics and fusion.

Similar to some vacuole-inhabiting bacteria*, Chlamydia* also subverts the endocytic pathway to create a replicative niche. *Chlamydia* is also a very proficient modulator of the host cytoskeleton through complex interactions of its secreted effectors with the host cell. This manipulation is even more interesting when considering that *Chlamydia* has a relatively small genome for bacteria (1.04 and 1.23 Mb for *C. trachomatis* and *C. pneumoniae* respectively) and relies on the host for their metabolic requirements (Stephens et al., 1999). Furthermore, ∼10% of its genome encodes for virulence effectors (Betts-Hampikian and Fields, 2010) which, as for some other intracellular pathogens, are delivered through specialized secretion systems. Similar to *Legionella*, the *Chlamydia-*containing inclusion (the term used for the replicative vacuole) is diverted from the endocytic pathway early on, and is rather trafficked to the microtubule organizing centre (MTOC) via dynein-mediated movement. From here, they are in an ideal position to intercept lipids and nutrient-rich exocytic vesicles. Markers for early endocytic- and late endocytic-compartments are absent from the inclusion (such as Rab5, Rab7 and LAMP-1) (Rzomp et al., 2003; Scidmore et al., 2003). However, several other Rab GTPases are recruited to the inclusion, such as Rab1, -4, -6 (*C. trachomatis* only), -10 (*C. pneumoniae* only), -11 and -14 (Rzomp et al., 2003; Capmany and Damiani, 2010). The recruitment of the different Rab GTAPases is important for the modulation of fusion events, for example the prevention of lysosomal fusion and promoting of fusion with lipids and nutrient-rich exocytic vesicles. *Chlamydia* further modulate vesicle fusion via interaction with SNARE proteins.

Subversion of the canonical phagocytic pathway by *Salmonella* uses similar mechanisms to that of, both, the vacuole-residing bacteria and those opting for a cytosolic lifestyle. After internalization, *Salmonella* remains in a modified phagosome – the *Salmonella* containing vacuole (SCV). Similar to the microbes already mentioned, *Salmonella* utilize secretory systems to deliver their effectors to the host and have two T3SS encoded on different pathogenicity islands (SPI-1 and SPI-2) (LaRock et al., 2015). The early effectors secreted by *Salmonella* (via T3SS-SPI1) are important for the establishment of this early SCV. Shortly after being phagocytosed SCV associates with early endosome markers EEA-1 and Rab5 and via its effector SopB (a phosphatase), delays lysosome fusion by indirectly preventing Rab GTPases from binding to the phagosomal membrane (Steele-Mortimer et al., 1999; Mallo et al., 2008; Bakowski et al., 2010). Recruitment of sorting nexins (SNX) help in the progression of SCV maturation, SNX1 specifically induces tubulation and is involved in the removal of the cation-dependent mannose-6-phosphate receptor (MPR) that may be important for the lack of lysosomal enzymes in the late SCV (Bujny et al., 2008). Additionally, SNX3 transiently interacts with the early SCV and is required for tubule formation and recruitment of late endosomal markers Rab7 and LAMP-1 (Braun et al., 2010). The replacement of early markers at this stage is accompanied by a decrease in both bacterial cytoplasmic and SCV pH (Chakraborty et al., 2015). This drop in pH is crucial for induction of SPI-2 genes required for subsequent effector secretion. The effectors secreted by T3SS-SPI-2 change the early SCV into a late SCV that is uniquely suited for bacterial replication. Examples of SPI-2 effectors involved in SCV maturation include SifA and SopD2. SifA complexes with SifA-and-Kinesin-Interacting-Protein (SKIP). The SifA-SKIP complex sequesters and binds Rab9, thereby inhibiting Rab9-dependent recruitment of MPR (McGourty et al., 2012). SopD2 impairs the Rab7-dependent recruitment of RILP and FYCO1 (FYVE and Coiled-coil domain Containing protein 1). RILP and FYCO1 are involved in vesicular trafficking along microtubules and indirect inhibition of their recruitment by SopD2 delays delivery of the SCV to lysosomes (D’Costa et al., 2015). At this stage, the SCV is similar to a late endosome (with markers LAMP-1, Rab7 and V-ATPase), but not enriched with lysosomal enzymes, possibly due to the lack of MPR and incomplete lysosome fusion (McGourty et al., 2012). Similar to *Chlamydia, Salmonella* exploit dynein-mediated transport (via its effectors) to arrive at a juxtanuclear position near the microtubule organizing center (MTOC). At this location, *Salmonella* distinguishes itself from other intracellular pathogens with the formation of a dynamic tubular network composed of *Salmonella* induced filaments (SIFs) (Knuff and Finlay, 2017). SIFs are required for SCV integrity, enabling continuous fusion of host vesicles to SCV and are associated with late endosomal markers such as LAMPs, Rab7, V-ATPase, cholesterol and lysobisphosphatidic acid (LBPA), as well as low levels of MPR and cathepsin D. Furthermore, another similarity with other vacuole-living bacteria, is the communication between the SCV and the ER, illustrating the extensive interactions of SIFs/SCV with the host cell (Santos et al., 2015). However, unlike the other vacuolar bacteria’s interaction with ER-derived components, the *Salmonella* SCV interaction with the ER-derived coat protein complex II (COPII) can result in SCV rupture and *Salmonella* hyper-replication in the cytosol (Santos et al., 2015).

In comparison to the more meticulous modulations mentioned, *Listeria monocytogenes* takes a relatively more radical (and perhaps destructive) approach to ensure intracellular survival. Manipulation of the clathrin-mediated endocytic pathway facilitates entry into non-phagocytic cells (Veiga and Cossart, 2005), whereas entry into macrophages is achieved via phagocytosis and initial engulfment of bacteria to form phagosomes. However, with the help of the cholesterol-dependent pore forming toxin listeriolysin-O (LLO), phagosome-lysosome fusion is disrupted via dysregulation of pH and calcium gradients across the phagosome membrane (Henry et al., 2006; Shaughnessy et al., 2006). Additionally, with the help of two phospholipases (PlcA and PlcB), LLO promotes escape of the bacteria from phagosomes into the cytosol (Mitchell et al., 2015; Smith et al., 1995). Once in the cytosol, bacteria undergo rapid growth and subsequently hijack the host’s actin polymerization machinery to move within the cytosol and ultimately spread in a cell-to-cell manner (Mitchell et al., 2015; Skoble et al., 2017). Although not as intricate as *Brucella* and *Legionella*, *Listeria* is also capable of slow replication in macrophage vacuoles (instead of rapid cytosol replication) via the formation of spacious *Listeria*-containing phagosomes (SLAPs) (Birmingham et al., 2008). SLAP formation is dependent on LLO, but unlike phagosome rupture observed with cytosolic life, intermediate LLO expression is required for interference with phagosomal pH, without phagosomal rupture. Bacteria containing SLAPs are LAMP-1^+^, which indicates that these are endocytic compartments. However, no drop in pH is observed, due to LLO-mediated uncoupling of pH gradients across the membrane and prevention of lysosome fusion. Furthermore, SLAP formation is dependent on autophagy and is hypothesized to be triggered by the damage caused to phagosomes by LLO.

The opportunistic pathogen *Cryptococcus neoformans* (Cn) is also capable of infecting and replicating at high numbers in macrophages and may possibly utilize these phagocytes as shuttle for their dissemination across the blood brain barrier. An important virulence factor of Cn is its capsule, which ensures survival by protecting against phagocytic uptake and oxidative stress, once infiltrated into the host circulation (Zaragoza et al., 2008; Bojarczuk et al., 2016). However, phagocytosis can be triggered by direct recognition of Cn capsule components or indirectly via complement (Johnston and May, 2013). After Cn internalization by macrophages, it resides in phagosomes which mature into a phagolysosome, as usual. Interestingly, this microbe does not seem to radically modulate the phagosomal maturation process, but rather seems able to thrive at the lower pH of the maturing phagosome. Some early- and late-endosomal markers are present on these phagosomes, including EEA-1, Rab5, Rab11, MPR, LAMP-1 and cathepsins, with live Cn inducing premature removal of Rab5 and Rab11 from the Cn phagosome, which may influence phagosome acidification (Tucker and Casadevall, 2002; Davis et al., 2015; Smith et al., 2015). The phagolysosomes still acidify, but final pH is maintained slightly higher, at around 5.3 (vs. normal phagolysosome pH of ∼4.5), which is the optimal pH for Cn growth. Additionally, damage to the phagolysosome membrane favors Cn survival and possibly contributes to the slight increase in pH observed with live Cn (Davis et al., 2015). Recently urease activity was found to influence phagosomal pH, which through production of urease-derived ammonia can increases pH (Lerm et al., 2017; Fu et al., 2018). Furthermore, membrane damage to the phagolysosome results in permeabilization of the membrane and subsequent leakage of lysosomal enzymes (e.g., cathepsins), the loss of which may also increase survival of Cn within the phagolysosome (Wozniak and Levitz, 2008). Furthermore, the release of these enzymes, can result in activation of inflammasomes and subsequent cell death (Chen et al., 2015). It is clear that Cn is capable of modulating phagosome maturation to some extent, but the search for responsible effectors is still ongoing.

These studies illustrate how some pathogens manipulate the phagocytic process in seemingly divergent ways to reach an identical end goal of intracellular survival. In doing so they ensure their own propagation and dissemination to elicit disease. Importantly, in our opinion, this demonstrated susceptibility to manipulation of the phagocytic process supports the feasibility of drug delivery systems that harness one or more of the microbial strategies presented here. Although there is still much research to be done on the exact microbial effectors involved in manipulation of the endocytic pathway, the available literature can already be used to make informed decisions as to which effectors can be used in the development of autologous drug delivery systems.

In the context of a complete macrophage-based drug delivery system, the manipulation of the endocytic pathway for retention and protection of cargo is only the first step. The next step to consider in the development of an effective delivery system, is the expulsion of drug cargo from macrophage vehicles. To this end, the mechanisms used by microbes can again be mined and possibly exploited to achieve cargo expulsion.

### Expulsion From Host Cell

Turning attention now to the expulsion phase, which is a vital requirement for pathogenic dissemination of microorganisms and which can be induced by either the infected host cell, or by the pathogen itself. Some microorganisms utilize host cell machinery to facilitate their escape, while others induce either accidental or intended host cell death, resulting in their release from the cell as a “side-effect.” Many microorganisms have been identified to have the ability to egress via one or more methods and some effectors in this process have been identified. However, in terms of manipulation of egress through upregulation or elimination of these effectors, very little data is available and substantial experimental work is still required in this niche. This can be attributed, at least in part, to a large degree of redundancy. This degree of redundancy is also seen in the bacterial mechanisms employed to modulate phagosome maturation, which adds complexity to the process of identifying a controllable pathway. Below, we provide a summary of the current knowledge regarding microbial egress, with an integrated discussion of its potential for therapeutic application.

Probably the most obvious technique used, given the ability of many microbes to manipulate phagosomal pH for intracellular survival, is the manipulation of pH to induce host cell death. This technique has been described in some detail for Mtb, which stabilizes phagosomal pH at ∼6.2–6.5 by interfering with the V-ATPase complex (Sturgill-Koszycki et al., 1994; Seto et al., 2011; Queval et al., 2017). This raised pH level is a pre-requisite for the ESX-1 dependent rupture of the phagosome (Simeone et al., 2015). The ESX-1 (T7SS) secretory system secretes two effector proteins, namely EsxA and EsxB, which form a heterodimer and are secreted by Mtb in a co-dependent manner (Renshaw et al., 2002). EsxA has membrane permeabilizing properties and EsxB is thought to act as a chaperone to prevent degradation and/or premature lytic activity (de Jonge et al., 2007; Zhang et al., 2016). EsxA effects phagosome rupture and escape to the cytosol, while being aided by the cell wall lipid phthiocerol dimycocerosates (PDIM) (Augenstreich et al., 2017; Quigley et al., 2017). This lipid has been proposed to primarily aid in phagosomal rupture, resulting in increased numbers of cytosolic bacteria – which in turn induces host cell necrosis and ultimately Mtb dissemination (Augenstreich et al., 2017; Quigley et al., 2017).

Other bacteria have also been described to escape through host cell membrane rupture resulting in cell death, albeit achieved by slightly different techniques. *Brucella* for example replicates within host cells, dissociating into two phenotypes, namely a smooth and a rough type. The rough phenotype has cytotoxic activity which breaks down the cellular membrane and is essential for bacterial dissemination (Pei et al., 2006, 2014). In this way, *Brucella* egress and dissemination is achieved through caspase-2 mediated cell death (Chen and He, 2009; Chen et al., 2011). Furthermore, this mode of cell death results in a pro-inflammatory response and recruitment of additional macrophages – that can be infected - in further aid of *Brucella* dissemination (Pei et al., 2014). It has, however, been proposed that *Brucella* can disseminate via cell-to-cell spread using an autophagy related mechanism (Starr et al., 2012; Smith et al., 2016). The final phase of *Brucellosis* intracellular life cycle is the formation of an aBCV which results from the engulfment of rBCVs into autophagosome-like structures via an autophagic process (Starr et al., 2008, 2012). This transformation to an aBCV is an essential prerequisite for bacterial egress via cell-to-cell spread (Starr et al., 2012; Smith et al., 2016). Interestingly, the VirB T4SS has been implicated in *Brucella* release via cell death and cell-to-cell spread, although the bacterial effectors have not been identified (Pei et al., 2008; Smith et al., 2016). The different modes of dissemination are possibly due to differences in experimental conditions, such as bacterial strains and cell lines used. Different bacterial strains may have different effectors or altered expression profiles that may result in different post-replicative outcomes (i.e., cell death or cell-to-cell spread) and different cell lines will also react differently to secreted effectors.

*Legionella* can also be placed in the category of intracellular pathogens that escape through macrophage cell death. Once a replicative LCV is established inside the macrophage, the bacteria converts to a replicative form and multiplies within the enclosed LCV. At high multiplicities of infection and subsequent termination of replication *Legionella* exhibit contact-dependent cytotoxicity, resulting in formation of pores in the host cell membranes (Kirby et al., 1998; Bachman and Swanson, 2001). Initially pores are formed within the phagosomal membrane (of the LCV), resulting in release of bacteria into the cytosol. The cytosolic bacteria are then able to form pores within the plasma membrane, resulting in osmotic lysis and release of bacteria (i.e., necrosis) (Kirby et al., 1998; Alli et al., 2000). The importance of the Dot/Icm secretory system of *Legionella* for pore-formation mediated lysis, and specifically that of the small inner membrane protein, IcmT, has been illustrated (Molmeret et al., 2002). Interestingly, in primary protozoan host cells, *Legionella* is capable of non-lytic release. The bacterial effectors LepA and LepB have been shown to play a role in manipulating the amoeba hosts exocytic pathway for dissemination (Chen et al., 2004, 2007). These effectors may also play a role in *Legionella* release from human cells or phagosome maturation and can potential form part of an artificial microbe drug delivery system.

Literature indicates that *Salmonella* exits cells via several mechanisms, including programmed cell death and flagella-facilitated escape. For example, the SPI-1 effector SipB can act by inducing caspase-1-dependent pyroptosis in macrophages (Hersh et al., 1999; Li et al., 2016). Briefly, SipB binds to and activates caspase-1, resulting in the cleavage of pro-IL1β and its secretion (Hersh et al., 1999; Li et al., 2016). While this inflammatory response should result in elimination of *Salmonella*, the over-activation during infection results in release of large amounts of bacteria capable of infecting naïve recruited cells. SipB is also able to induce apoptosis in a caspase-1 independent manner involving activation of caspase-2, -3, -6, and -8 (Jesenberger et al., 2000). In addition to apoptosis and pyroptosis, *Salmonella* is also able to induce oncosis in macrophages (Sano et al., 2007). Oncosis is associated with macrophage swelling resulting in cell death, with *Salmonella-*induced oncosis characterized by F-actin dissociation. Subsequently the flagellated *Salmonella* escapes from oncotic macrophages via flagellar movement (Sano et al., 2007).

Although the methods presented here will effectively release intracellular cargo, the associated host cell death may significantly contribute to tissue damage and secondary inflammatory damage, which may further delay recovery of patients. While certainly an option to consider for further development, specifically where cell death and increased inflammation would not be as detrimental (e.g., cancerous tissue), a more optimal solution in scenarios where minimization of inflammation – as well as the availability of functional macrophages - might be more critical, would be achieving release of drugs without the sacrifice of host cells.

Manipulation of the host cell’s expulsion mechanics without killing the host has indeed been described for a few microbes, although much less information is available in this context. From the literature, it seems that only two egress methods have been described: direct spread into neighboring cells and expulsion into the extracellular environment. As briefly eluded to earlier, *Listeria monocytogenes* escapes from the host phagosome into the cytosol through activity of pore forming LLO (Alberti-Segui et al., 2007). However, complete escape from the host macrophage is aided by the effector responsible for modulation of the host actin polymerization machinery. Surface anchored actin assembly-inducing protein (ActA) interacts with the ARP2/3 complex to mediate actin polymerization on the bacterial surface, which in turn creates sufficient force to induce membrane protrusion and cell-to-cell spread (Skoble et al., 2017). The actin-propelled bacteria creating these membrane protrusions induce uptake into neighboring cells via a process called efferocytosis (Czuczman et al., 2014). Here, LLO damages the plasma membrane of protrusions, resulting in surface presentation of the inner membrane leaflet lipid, phosphatidylserine (PS) (Czuczman et al., 2014). The PS^+^ protrusions are recognized by the T cell immunoglobulin and mucin-domain containing protein 4 (TIM-4) on macrophages, which subsequently mediates the uptake of PS^+^ protrusions (Czuczman et al., 2014). The bacteria may also be present in PS^+^ vesicles, formed as a result of Ca^2+^ dependent membrane repair and scission of the initial PS^+^ protrusion (Czuczman et al., 2014). Both PS^+^ vesicles and protrusions are similarly taken up by neighboring cells via TIM-4. *Listeria* are one of the few phagocytically internalized microorganisms that allow host cell survival after escape. Thus, the processes regarding expulsion of *Listeria* is of great interest as target for manipulation or adoption in therapeutic drug delivery systems.

*Chlamydia* is also known to exit the host cell by extrusion, although it can also induce cell lysis (Hybiske and Stephens, 2007). These methods exhibit almost identical prevalence, but are markedly distinct and independent. The exact bacterial trigger facilitating either one or the other outcome has yet to be fully elucidated. Cell lysis is known to be protease and calcium dependant, and entails perforation of the inclusion body (*Chlamydia*-containing vacuole) and plasma membrane (Hybiske and Stephens, 2007). *Chlamydia*-infected host cell lysis has been suggested to be linked to microbe-associated apoptotic cell death, although minimal direct evidence exists in support of this notion (Perfettini et al., 2003). Regardless, the extrusion capability of *Chlamydia* from host cells without cell lysis is of greater interest here. With this technique, inclusion body extrusion requires actin polymerization, myosin II, RhoA and Neuronal Wiskott-Aldrich Syndrome Protein (N-WASP) (Hybiske and Stephens, 2007). The formation of an actin coat around the inclusion is correlated to extrusion out of the host cell. Host- and bacterium-derived factors play a role in the formation of the actin coat. For example, in humans, host-derived septins (GTP-binding proteins) form structures around the inclusion and co-localize with F actin, resulting in the formation of F actin fibers around the inclusion (Chin et al., 2012; Volceanov et al., 2014). This process facilitates normal extrusion of *Chlamydia* inclusions from host cells (Volceanov et al., 2014). Actin stabilization by jasplakinolide (actin polymerization agent) alone was reported to induce extrusion, which substantiates the role of septins in extrusion (Hybiske and Stephens, 2007). In terms of therapeutic application, this may suggest that the intervention achieved by *Chlamydia* on the host cell mechanics may be less detrimental to the host cell compared to other microbial exit strategies. In a therapeutic context, this may result in faster normalization of function in the host cell, which is much desired, as these host cells may then be able to participate in the normal inflammatory process that would be required for clean up after the drug has fulfilled its function.

Non-lytic release into the extracellular space has also been observed for *Cryptococcus neoformans*, albeit at low frequencies of 5–15% *in vivo* (Bojarczuk et al., 2016). Autophagy has been implicated in Cn intracellular lifestyle with components such as Atg-2a, -5, -9a, -12 and LC3 observed in close proximity to the Cn containing phagosome (Qin et al., 2011; Nicola et al., 2012). The effect of autophagy is dependent on opsonin, macrophage type and activation state (Nicola et al., 2012). Autophagy does seem to play a role in host defense against Cn, with disruptions in autophagy affecting host fungistatic activity and fungal growth (Qin et al., 2011; Nicola et al., 2012). However, this is a double edged sword with autophagy also seemingly playing a role in Cn release. This is evident by the observation of Atg-5-knockout clones of J774.16 and RAW264.7 cells having reduced incidence of non-lytic exocytosis events (Qin et al., 2011; Nicola et al., 2012). This, along with the observation of LC3 surrounded cells outside macrophages, suggests a possible role of autophagy in non-lytic release of Cn (Nicola et al., 2012). Therefore, while autophagy aids in the host defense against Cn, it also participates in the dissemination of Cn through non-lytic release. This eludes to a balance that must be maintained by the host with regard to autophagy, with either decreased fungistatic activity combined with decreased non-lytic release, or *vice versa.* In addition to the potential role of autophagy in the non-lytic release of Cn, other factors can also play a role in this route of Cn dissemination. The increase in pH of the Cn-containing phagosome results in increased occurrence of non-lytic release. Artificially increasing phagosomal pH results in increased expulsion of Cn and when compared to the *in vivo* situation, the damage caused by Cn to the phagosome could result in a similar pH increase (Nicola et al., 2011). Unfortunately, the Cn effectors responsible for non-lytic release have not yet been elucidated. However, in addition to the potential role of autophagy, the MAP extracellular receptor kinase 5 (ERK5) of host cells is implicated in the regulation of non-lytic release, with its inhibition resulting in increased release rates (Gilbert et al., 2017). Furthermore, actin polymerization also plays a role in release of Cn from the host (Johnston and May, 2010). In contrast to other pathogens such as *Listeria* and *Chlamydia*, actin polymerization inhibits Cn release through actin flashes on the Cn containing phagosome (Johnston and May, 2010). Although some information regarding the non-lytic release of Cn is available, it still remains poorly understood and the exact mechanism for this escape method is still elusive.

The body of research investigating intracellular pathogens and their host-interacting mechanisms is significant. However, specific information on the microbial effectors is still largely lacking, probably owing to the fact that the main focus of research was the *prevention* of these microbial actions, rather than full elucidation thereof for *implementation*. In addition, effectors secreted by invading microbial forces exhibit a large degree of built in redundancy, so that it is not surprising that the task of identifying specific roles for specific effectors has remained largely unaccomplished. Specifically, in terms of microbial expulsion, information is largely lacking, with only a handful of known microbial effectors. Considering this, clearly a new approach is required. The recent advancements in gene editing, heterologous expression, live cell imaging and -omics technologies may provide a more powerful platform from which to investigate the complex host-pathogen interaction and the effectors involved, especially in the context of expulsion from immune cell hosts.

## The Impossible Made Possible?

**FIGURE 2:**
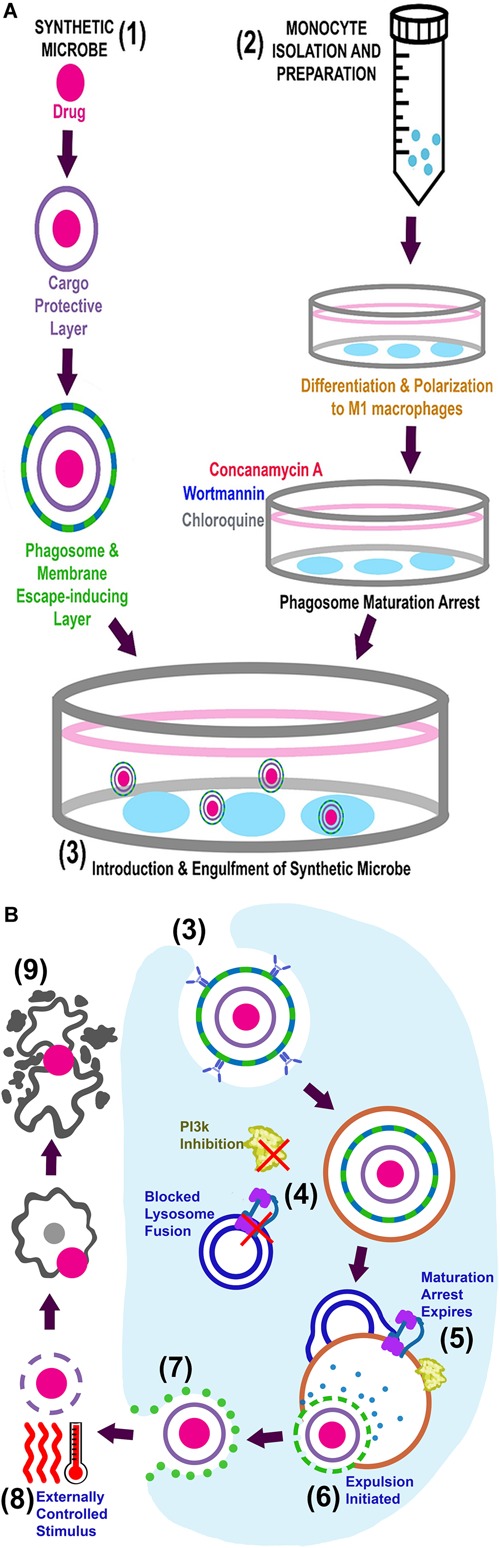
Visual representation of the proposed system. **(A)** Simultaneous preparation of the synthetic microbes (1) and autologous macrophages (2) are followed by introduction of the synthetic microbe into phagosome maturation arrested macrophages (3). The complete system can now be administered into circulation for *in vivo* delivery. **(B)** Intracellular events for *in vitro* engulfment of synthetic microbe (3), *in vivo* maintenance/expulsion (4–7) and final delivery of drug at target site (8–9).

From our review of the literature, we propose that most of the limitations of current drug delivery systems can be overcome by harnessing microbial strategies. We hypothesize that the synthetic microbe drug delivery system we describe here would (a) address poor drug-delivery to target tissue – especially at sites with low blood supply – (b) increase treatment efficacy with lower treatment doses and thereby (c) reduce adverse host reactions. A visual representation of the proposed system is provided in Figure 2.

In terms of the system we propose, we foresee two novel preparatory steps to be done in parallel. Firstly, monocytes should be isolated from peripheral blood collected from individuals with a requirement for drug delivery, to enable autologous re-infusion. These macrophages can then be propagated in culture, differentiating and polarizing them into M1 phenotype macrophages, as previously described (Mia et al., 2014), to achieve the phenotype known to be most capable of translocating out of circulation and into tissue (Arnold et al., 2007; Africa and Smith, 2015a,b). In order to prevent intracellular degradation, polarized macrophages can be treated to achieve transient phagosome maturation arrest, by *in vitro* exposure to arresting agents such as Wortmannin, Concanamycin A and Chloroquine, which we have previously successfully illustrated (Visser and Smith, 2018). The fact that phagosome maturation arrest is achieved by *in vitro* intervention is a strength of this model, as this eliminates patient exposure to these potentially harsh chemicals and their related adverse effects. Secondly, the drug should be packaged in multiple layers of nanoparticle coatings (Figure 2A). These layers will serve to protect the drug from degradation, and can be peeled away in sequential, controlled steps to achieve controlled expulsion and release of the drug after delivery at its required site. With advancements in polymer sciences, smart polymers can be designed that have effectors conjugated to the polymers and can also be developed for directed multiphasic release of effectors at certain checkpoints. (The nature of the different layers will be clarified in the discussion of the expulsion phase and illustrated in Figure 2).

To construct the synthetic microbe drug delivery system, phagosome maturation arrested macrophages will engulf opsonized nanoparticle-enclosed drugs (the synthetic microbe) and form a phagosome containing this cargo (Figure 2). The number of synthetic microbes taken up per macrophage may be optimized by adjusting macrophage-microbe exposure time. Uptake is largely dependent on particle proximity to the macrophage as well as size and shape of particles (Beningo and Wang, 2002; Champion and Mitragotri, 2006; Underhill and Goodridge, 2012). Determining the exact onset of engulfment, following introduction of particles into cell media, is complex and may need optimization on a case-specific basis. Unpublished data from our group suggests initiation of engulfment to occur within 5 min, with particles included at a 1:4 ratio with cells. From the literature, the last stage of phagosome maturation (phagolysosome biogenesis) occurs around 1 h after engulfment (Jahraus et al., 1998). Our implementation of an arbitrary exposure time of 1 h resulted in significant 4.5 μm particle engulfment (>1 particle per cell, in ∼70% of cells) by both arrested and untreated macrophages. Since macrophages are capable of repeated engulfment cycles (irrespective of arrest), carrier cells should be removed from drug-containing media and washed after the appropriate engulfment period, to prevent overloading of macrophages and potentially compromising their ability to traverse endothelial barriers, as well as eventual cell death (if particle load exceeds cytosol volume or steric hindrance causes membrane rupture during transmigration) (Champion and Mitragotri, 2006; Visser and Smith, 2018). At this point, the drug-loaded macrophages are ready for autologous re-administration into the patient via infusion into the peripheral circulation (Figure 2B).

As stated earlier, macrophage migration to sites for delivery will rely on the inherent macrophage capacity for chemotactic mobility. However, *in vivo* testing of the system will elucidate whether further optimization is required. Upon arrival at the site for delivery, controlled expulsion of the cargo and subsequent release of the drug will be achieved through multiple consequential steps. Specifically, the phagosome lysing and membrane escaping agents (i.e., microbial effectors) would be released upon arrival at target location. This can only be achieved after transient phagosome maturation has expired and phagolysosomal degradation agents activate this layer. This biphasic layer would, in turn, allow escape of cargo into the cytosol, as well as expulsion thereof into the extracellular environment through microbial effectors. The substantial amount of genome data currently available should enable relatively comprehensive genomic data mining for novel and existing microbial effectors. The use of genome mining in this context will only be useful if information (e.g., homology regions, structural information) about effectors are available. The use of techniques such as proteomics along with bioinformatic mining/analysis has been suggested as more useful tools in combination to identify effectors (Na et al., 2015; Cheng et al., 2017). For example, in recent studies, proteomics have been used to identify novel effectors in *Salmonella*. Although proteomics can be a useful tool in identification of effectors it is only the first step. Next would be to heterologously express the identified effectors. Several bacterial effectors have been expressed in heterologous systems with success (Churchill et al., 2005; Skoble et al., 2017; Weigele et al., 2017). Most notably, the *Listeria* effectors LLO and ActA have been recombinantly expressed, with recombinant LLO also being used for the effective delivery of several compounds to the cytosol of cells (Lee et al., 1996; Mandal and Lee, 2002; Provoda et al., 2003; Stier et al., 2005; Skoble et al., 2017). The ability to identify and express possible effectors creates the opportunity to manipulate the host endocytic pathway. However, these effectors need to be protected from degradation and secreted in a sequential manner to effectively modulate host processes.

In order to prevent asynchrony of final drug release and arrival of synthetic microbe at the target site (i.e., release of drug at inappropriate sites), an innocuous, bio-compatible layer, such as PNIPAAm (mentioned above), could be incorporated. This layer would be the inner most layer and protect the drug until controlled drug dissemination at the required site is achieved via external stimulus (e.g., temperature or ultrasound). In the case of a PNIPAAm layer, final drug dissemination can be achieved by temperature increase (Feng et al., 2014). In addition, microbubbles could also be incorporated as protective drug micelle, instead of PNIPAAm. These microbubbles would then be degraded by external ultrasound stimulation (Huang et al., 2015; Lv et al., 2016; Fan et al., 2018). This layer should ideally consist of non-biodegradable but bio-compatible constituents, to prevent drug release into circulation and exposure to off-target tissue. Furthermore, augmented host protection can be achieved by allowing the safe extrusion of intact drug-micelles through the urinary tract.

The redundancy employed in the aforementioned approach is another characteristic that could be adopted from the plethora of redundancy employed by microbes during immune evasion, in order to maximize control over drug delivery and minimize risk of undesired effects.

## Post-Delivery Clearance of the System

Given the potential complication of polymer accumulation *in vivo*, or premature drug release, it is perhaps relevant to briefly discuss safety aspects of this system. The utilization of M1 macrophages would be advantageous as they would remain at target locations post-delivery and could potentially contribute to faster resolution of either primary inflammation (present due to the disease state) or secondary inflammation (required to clear bacterial debris after effective treatment). In support of this theory, the M1/M2 phenotype presents with some plasticity as tissue resident macrophages have been shown to change phenotype following appropriate effector exposure or systemic infection and activation by pathogens (Arnold et al., 2007; Mills, 2012). This low risk feature is the result of the non-lytic mechanisms employed to achieve expulsion, which is novel.

In terms of clearance of the components of the nanoparticle layers themselves, polymeric nanoparticles have demonstrated clearance rates of hours to several days (Alexis et al., 2008; Feng et al., 2014). Macrophages failing to reach their intended target sites may still release the encapsulated drug, but final dissemination of drugs at unintended sites is unlikely, due to the requirement for external stimulus. Thus, encapsulated drugs, polymeric debris and loaded macrophages should be efficiently cleared from the system, so that no long-term implications due to residual drugs or synthetic microbe components are expected.

Clearly, a model proposing quite significant paradigm shifts, such as the one we described here, requires substantial *in vitro* and *in vivo* testing and optimization by multiple laboratories. Given the requirement for *in vivo* tracking of cells, a zebrafish model may be the ideal choice for pilot studies, as the basic anatomy of the fish facilitates leukocyte migration tracking (Langenau et al., 2004) and this model has been employed in the context of macrophage migration (Wang et al., 2016; Barros-Becker et al., 2017). Additionally, *in vivo* tracking can also be achieved by permanent intracellular cell labeling and/or lasing techniques (Schubert et al., 2015) that would elucidate the exact location of carrier macrophages as well as their cargo over an extended period of time.

## Conclusion

Intracellular pathogens have developed a unique arsenal of tools to evade the immune system and thrive within host cells. The modulation of host processes, specifically modulation of the endocytic pathway, not only make these pathogens difficult to treat and dangerous, but also provides the opportunity to utilize and modify their methods for human gain. If the right combination of these effectors are repurposed, they can be used to develop a macrophage-based delivery system for the transport and controlled delivery of therapeutic agents packaged into a “synthetic microbe” as described here, to significant benefit of patients currently struggling with diseases at non-accessible sites or those caused by multi-drug resistant pathogens.

The success of a host-derived or biological delivery system is dependent on a key understanding of how microbial effectors work and what combination would result in effective release without severe side-effects, keeping in mind that the effectors are indeed potent virulence factors. Advancements in molecular biology, -omics, bioinformatics and live cell imaging have resulted in the identification of effectors and their roles in the host-microbe/pathogen interaction, using and building on this information, effectors can be chosen that would result in the desired outcome. Utilizing synthetic biology and heterologous expression, effectors can be produced and tailored for specific functions. Although some developmental steps for the proposed synthetic microbe drug delivery model remains to be addressed in more detail, we believe that rapid development in e.g., polymer design and the aforementioned advancements in techniques that are used to characterize and tailor effectors, can be used to overcome these limitations in the very near future.

## Author Contributions

All authors listed have made a substantial, direct and intellectual contribution to the work, and approved it for publication.

## Conflict of Interest Statement

The authors declare that the research was conducted in the absence of any commercial or financial relationships that could be construed as a potential conflict of interest.
